# CD146 as an adverse prognostic factor in uterine sarcoma

**DOI:** 10.1186/s40001-015-0160-2

**Published:** 2015-08-21

**Authors:** Yun Zhou, He Huang, Lin-Jing Yuan, Ying Xiong, Xin Huang, Jia-Xin Lin, Min Zheng

**Affiliations:** Department of Gynecology Oncology, Sun Yat-sen University Cancer Center, Guangzhou, People’s Republic of China; State Key Laboratory of Oncology in South China, Guangzhou, People’s Republic of China; Collaborative Innovation Center for Cancer, 651 Dongfeng Road East, Guangzhou, 510060 Guangdong People’s Republic of China

**Keywords:** Uterine sarcoma, CD146 overexpression, Prognostic marker

## Abstract

**Background:**

Uterine sarcoma is an aggressive malignancy with a poor prognosis. This study aimed to determine the expression of CD146, P53, and Ki-67 in uterine sarcoma and to evaluate their prognostic significance.

**Methods:**

We retrospectively analyzed the prognosis and clinicopathologic features of 68 patients with uterine sarcoma. Immunohistochemical analyses of CD146, P53, and Ki-67 were performed in tissue samples collected from these patients and their relationship with prognosis was investigated.

**Results:**

The 5-year overall survival (OS) rate was 46 %. Endometrial stromal sarcoma (ESS) patients had a better prognosis than leiomyosarcoma (LMS) patients, with a 2-year survival rate of 82 %. The membrane and cytoplasm of tumor cells exhibited CD146 overexpression in 8 (32 %) ESS cases, which was less than the 25 (69.4 %) cases observed in LMS and 2 (28.6 %) in MMMT. CD146 overexpression in the membrane and cytoplasm of tumor cells was closely related to lymph node metastasis (*P* = 0.021) and Ki-67 overexpression (*P* = 0.0053); there was no significant correlation with age, tumor size, International Federation of Obstetrics and Gynecology stage, or P53 overexpression in LMS.

**Conclusions:**

CD146, P53, and Ki-67 are overexpressed in uterine sarcoma. CD146 expression correlates with lymph node metastasis and is associated with poor OS in LMS; it may be a potential prognostic marker for LMS.

## Background

Uterine sarcoma, a rare and heterogeneous malignant tumor, accounts for only 3 % of all uterine malignancies [1]; the annual incidence is <2 per 100,000 women [2]. According to the World Health Organization (WHO) 2003 classification system, there are three main uterine sarcoma subtypes: leiomyosarcoma (LMS), endometrial stromal sarcoma (ESS), and undifferentiated sarcoma (UD) [3]. Carcinosarcoma (malignant mixed Müllerian tumor, MMMT) is an undifferentiated sarcoma that was excluded as a uterine sarcoma in the 2009 National Comprehensive Cancer Network guidelines; however, most retrospective studies still include MMMT as a uterine sarcoma [4]. The prognostic factors of uterine sarcoma are not well established, with no consensus on their significance.

*P53* gene mutations occur in approximately one-half of all tumors in a wide range of human malignancies and overexpression is associated with poor prognosis [5]. There are several reports of P53 overexpression in uterine LMS, MMMT, and ESS [6, 7]. Ki-67 expression is also associated with poor prognosis in uterine sarcoma [8]. During the last decade, several studies were performed to assess estrogen receptor, progesterone receptor, P53, and Ki-67 expression as prognostic factors in LMS [9].

CD146, an adhesion molecule belonging to the immunoglobulin superfamily, was first identified as a melanoma-specific cell adhesion molecule [10]. CD146 is overexpressed in cancers such as epithelial ovarian cancer and breast cancer, in addition to melanoma [11, 12]. A recent study suggested that CD146 might be involved in tumor development and be associated with prognosis in several systemic tumors [13].

Previous studies have shown that CD146 is a tumor angiogenesis marker. Zhang et al. reported that CD146 is present in most blood vessels in cervical and endometrial cancer, suggesting that it may be actively involved in cervical and endometrial cancer metastasis via the vascular system [14]. The anti-CD146 monoclonal antibody AA98 inhibits angiogenesis and tumor growth. AA98 has antiangiogenic properties in vitro and in vivo. AA98 inhibits human umbilical vein endothelial cell proliferation and migration as well as angiogenesis, validating CD146 as a novel target for antiangiogenic agents [15]. Wen et al. found that CD146 expression promoted epithelial–mesenchymal transition, correlating with mesenchymal marker expression and indicating poor prognosis in gastric cancer. CD146 downregulation induced downregulation of the genes for vimentin, fibronectin, and thrombospondin, which are mesenchymal markers [16].

However, no study to date has reported on CD146 expression in uterine sarcoma. In the present study, we compiled data from patients diagnosed with uterine sarcoma from the Sun Yat-Sen University Cancer Center (SYSUCC) in Guangzhou, China to investigate associations between the expression of CD146, the cell proliferation molecule Ki-67, and P53 and the patients’ clinicopathologic features and prognosis.

## Methods

### Patient selection and treatment

This retrospective study involved 68 patients diagnosed with primary uterine sarcoma who underwent surgical treatment from January 1996 to December 2011 at SYSUCC. Paraffin-embedded (PE) tissue samples were collected from the pathology department as stored specimens. Prior consent from patients was obtained for research use of their PE tissues. This study was approved by the SYSUCC Institutional Review Board. We agreed to provide copies of the appropriate documentation if requested.

Patients were examined at 2-month intervals for the first 2 years, at 6-month intervals for the next 3 years, and once per year thereafter. The median follow-up period was 63.3 months (range 1–272.1 months). The primary end point was any cancer-related death. To ensure diagnostic accuracy, experienced pathologists conducted the pathologic diagnoses. Clinical data were retrieved from medical records in the institutional database. The follow-up data were updated in August 2013.

The patients were selected using the following criteria: diagnosed with uterine sarcoma according to the 2003 WHO diagnostic criteria; no preoperative antitumor treatment; no previous malignant disease or second primary tumor; and complete clinicopathologic and follow-up data available. Patients who met these criteria were included.

The patients’ clinicopathologic characteristics were: age at diagnosis; International Federation of Obstetrics and Gynecology (FIGO) stage; histopathologic subtype; surgical procedure; postoperative adjuvant therapy; pelvic lymphadenectomy; lymph node involvement; tumor size; distant metastasis and recurrence; and CD146, P53, and Ki-67 immunohistochemical staining patterns.

The standard surgical treatment for ESS and LMS was total abdominal hysterectomy (TAH) plus bilateral salpingo-oophorectomy (BSO); pelvic lymphadenectomy (PL) was performed for late-stage patients (stage III/IV). Para-aortic lymph node dissection was performed for patients with suspected common iliac or para-aortic lymph node involvement. Omentectomy was performed for stage III/IV patients. Adjuvant radiotherapy and/or adjuvant chemotherapy was administered to patients with the following pathologic risk factors: incomplete resection in late-stage cases; positive pelvic lymph nodes; vascular and lymphatic permeation; high-grade ESS; tumor size >5 cm; and mitotic index (MI) >10/high-power field (HPF). Radiotherapy comprised external pelvic irradiation (18 MV X-rays) using the multiport technique. Daily fractions of 1.8–2.0 Gy were administered over 5–6 weeks for a total dose of 50 Gy. Chemotherapy mainly comprised cisplatin (60–75 mg/m^2^, d1) plus adriamycin (40–50 mg/m^2^, d1) plus dacarbazine (200 mg/m^2^, d1–5) as the primary drugs and was administered in three to five courses over a 3-week period.

The surgical treatment for MMMT usually comprised TAH plus BSO, PL, and omentectomy. We administered radiotherapy and chemotherapy for all MMMT cases.

### Immunohistochemistry

Formalin-fixed and PE tumor tissue samples were used for immunohistochemical analysis. Sections (4 μm thick) containing the area of the tumor with the highest grade of atypia and the least necrosis were obtained from the paraffin block, mounted on slides, and deparaffinized in xylene, ethanol, and distilled water. Following 10-min incubation with 3 % hydrogen peroxide in methanol, the slides were boiled in citrate buffer solution (pH 6) for 10 min using a domestic pressure cooker. The sections were incubated overnight at 4 °C with P53, P27, Ki-67, and CD146 antibodies (1:100; Zhongshan Golden Bridge Biotechnology, Beijing, China). Then, the slides were washed with phosphate-buffered saline and incubated for 30 min at 37 °C with biotinylated goat antimouse secondary antibody (1:25; Lab Vision, Fremont, CA, USA). All slides were stained with 3,3′-diaminobenzidine and scored for immunoreactivity based on the percentage of positive tumor cells (percent positivity) and staining intensity (weak, moderate, strong). Slides with scores of (−) or (+) were deemed negative; slides with scores of (++) or (+++) were deemed positive. Two experienced pathologists confirmed the results in a double-blind analysis [12].

### Statistical analysis

Statistical analyses were performed using SPSS v16.0 software (IBM, Armonk, NY, USA). Prognosis was determined based on overall survival (OS) and disease-free survival (DFS). OS was calculated from the time of histopathological diagnosis to the time of death or last recorded event. DFS was calculated from the time of surgical resection to the first evidence of recurrence or death from any cause. Life tables were used to calculate the survival rates and median survival time. The relationship between CD146 expression and clinicopathologic characteristics was assessed using Pearson’s Chi-square test. Survival curves were obtained using the Kaplan–Meier method. Log-rank tests were used for statistical comparison of curves. Univariate and multivariate Cox regression methods were adapted to evaluate potential prognostic factors, yielding hazard ratios and 95 % confidence intervals. *P* < 0.05 was considered statistically significant.

## Results

### Demographic characteristics

Table 1 summarizes the demographic characteristics of the patients. The mean age was 48.3 years; the median age was 47.5 years (range 20–81 years). Primary symptoms included irregular menstrual bleeding (19.2 %), postmenopausal or abnormal vaginal bleeding (30.8 %), pelvic mass (11.5 %), and abdominal pain (15.4 %). Twelve cases (17.6 %) were diagnosed incidentally. Of the 68 patients, 36 (52.9 %) had LMS, 25 (36.8 %) had malignant ESS, and 7 (10.3 %) had MMMT. Thirty-six cases (52.9 %) were stage I, 3 (4.4 %) were stage II, 22 (32.4 %) were stage III, and 7 (10.3 %) were stage IV.

**Table 1:** Patients’ clinical and histopathological characteristics (*n* = 68)

Characteristics	No. case	LMS (*n* = 36)	ESS (*n* = 25)	MMMT (*n* = 7)
Age (years)				
<50	38 (55.8 %)	20	17	1
≥50	30 (44.2 %)	16	8	6
Stage				
I	36 (53.9 %)	19	13	4
II	3 (4.4 %)	3	0	0
III	22 (32.4 %)	7	12	3
IV	7 (10.3 %)	7	0	0
Tumor size (cm)				
<5	30 (44.1 %)	16	11	3
≥5	38 (55.9 %)	20	14	4
Pelvic lymphadenectomy				
Yes	34 (50 %)	17	13	4
No	34 (50 %)	19	12	3
Lymph node metastasis				
Positive	24 (35.3 %)	18	4	2
Negative	44 (64.7 %)	33	9	2
Surgical procedure				
TAH ± BSO/OT/PL/PLND	8 (11.8 %)	4	4	0
TAH + BSO ±/PL/OT	29 (42.6 %)	17	12	0
TAH + BSO + PL ± OT ± PLND	31 (45.6 %)	15	9	7
Chemotherapy	22 (32.4 %)	13	9	0
Radiotherapy	4 (5.9 %)	1	3	0
Hormone therapy	1 (1.5 %)	0	1	0
Chemotherapy and radiotherapy	20 (29.4 %)	10	3	7
Chemotherapy and hormone therapy	3 (4.4 %)	1	2	0
Radiotherapy and hormone therapy	1 (1.5 %)	0	1	0
No adjuvant therapy	17 (25 %)	11	6	0
Distant metastasis and recurrence				
Pelvic	10 (14.7 %)	6	3	1
Pelvic and liver/lung	6 (8.8 %)	4	2	0
Lung	4 (5.9 %)	3	1	0
Other parts	6 (8.8 %)	5	1	0
None	42 (61.5 %)	18	18	6

*TAH* total abdominal hysterectomy, *BSO* bilateral salpingo-oophorectomy, *OT* omentectomy, *PL* pelvic lymphadenectomy, *PLND* para-aortic lymph node dissection

All patients underwent surgery as initial treatment: 8 (11.8 %) underwent TAH, 29 (42.6 %) TAH + BSO, and 31 (45.6 %) TAH + BSO + PL. Fifty-one patients (75 %) received postoperative adjuvant therapy. Lymphovascular space invasion (LVSI) and lymph node metastasis were observed in 24 patients (35.3 %). Twenty-six patients (38.2 %) developed recurrence and 25 patients (36.8 %) died during follow-up.

### CD146, P53, and Ki-67 expression and their relationship with clinicopathologic characteristics of uterine sarcoma

**Fig. 1:**
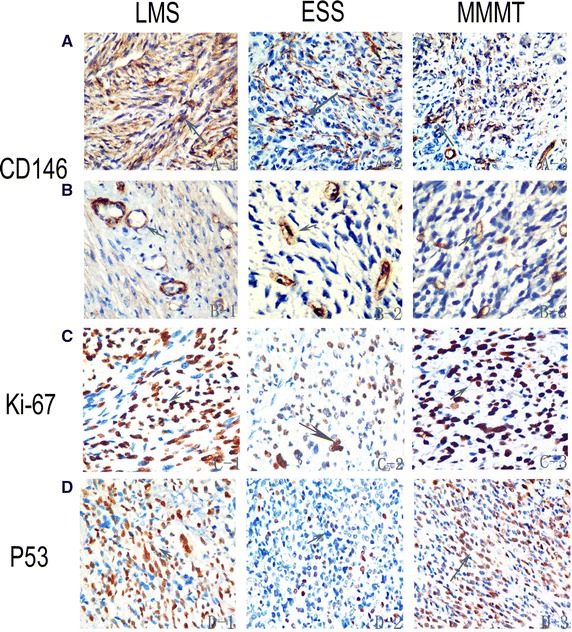
Immunohistochemical staining for CD146, Ki-67, and P53 expression in LMS, ESS, and MMMT. **A** CD146 mainly expressed in the epithelial compartment. **B** CD146 mainly expressed in the vascular epithelial compartment. **C** Nuclear Ki-67 immunoreaction. **D** Nuclear P53 immunoreaction

On immunohistochemical analysis of the uterine sarcoma tissue specimens, CD146-positive staining was localized not only in the epithelial compartment (the membrane and cytoplasm of tumor cells) but also in the vascular compartment. CD146 expression occurred more often in the vascular compartment. Forty-nine cases (72 %) exhibited vascular compartment CD146 expression, 35 of which were also positive for CD146 staining in the epithelial compartment (Fig. 1).

Overexpression of CD146 in the membrane and cytoplasm of tumor cells was observed in 8 (32 %) ESS cases, which was less than the 25 (69.4 %) cases observed in LMS and 2 (28.6 %) in MMMT. Positive nuclear P53 and Ki-67 immunoreactions were observed in 30 (44.1 %) and 48 (70.6 %) cases, respectively. ESS patients had the lowest positivity rates for both P53 and Ki-67 immunoreaction, with P53 and Ki-67 expressed in seven (28 %) and six (24 %) cases, respectively (Table 2).

**Table 2:** Immunohistochemical analysis of uterine sarcoma specimens

	CD146^a^	CD146^b^	P53	Ki67
LMS	25/36	32/36	22/36	24/36
ESS	8/25	12/25	7/25	6/25
MMMT	2/7	5/7	1/7	4/7

*ESS* endometrial stromal sarcoma, *LMS* leiomyosarcoma, *MMMT* malignant mixed Müllerian tumor

^a^Cytoplasm and membrane staining

^b^Vascular endothelial cell staining

We analyzed the correlation between CD146 expression status and the clinicopathological characteristics of LMS; the results are summarized in Table 3. CD146 overexpression on membrane and cytoplasm staining was closely related to lymph node metastasis (*P* = 0.021) and Ki-67 overexpression (*P* = 0.0053), but there was no significant correlation with age, tumor size, FIGO stage, or P53 overexpression.

**Table 3:** Correlation between CD146 expression and clinicopathologic features in LMS

	CD146 (−)	CD146 (+)	*P* value
Age			
<50	6	12	0.6
≥50	5	13	
Stage			
I–II	7	11	0.4
III–IV	4	14	
Tumor size (cm)			
<5	14	8	0.16
≥5	12	17	
LN statue			
Positive	2	15	0.021
Negative	9	10	
P53 expression			
Positive	7	15	0.68
Negative	4	10	
Ki67 expression			
Positive	4	20	0.0053
Negative	8	5	

*LN* lymph node

### Relationship between clinicopathologic parameters and survival

The median survival time was 30.7 months (range 1–271 months). The 2- and 5-year OS rates were 57 and 46 %, respectively; the 2- and 5-year DFS rates were 51 and 44 %, respectively.

**Fig. 2:**
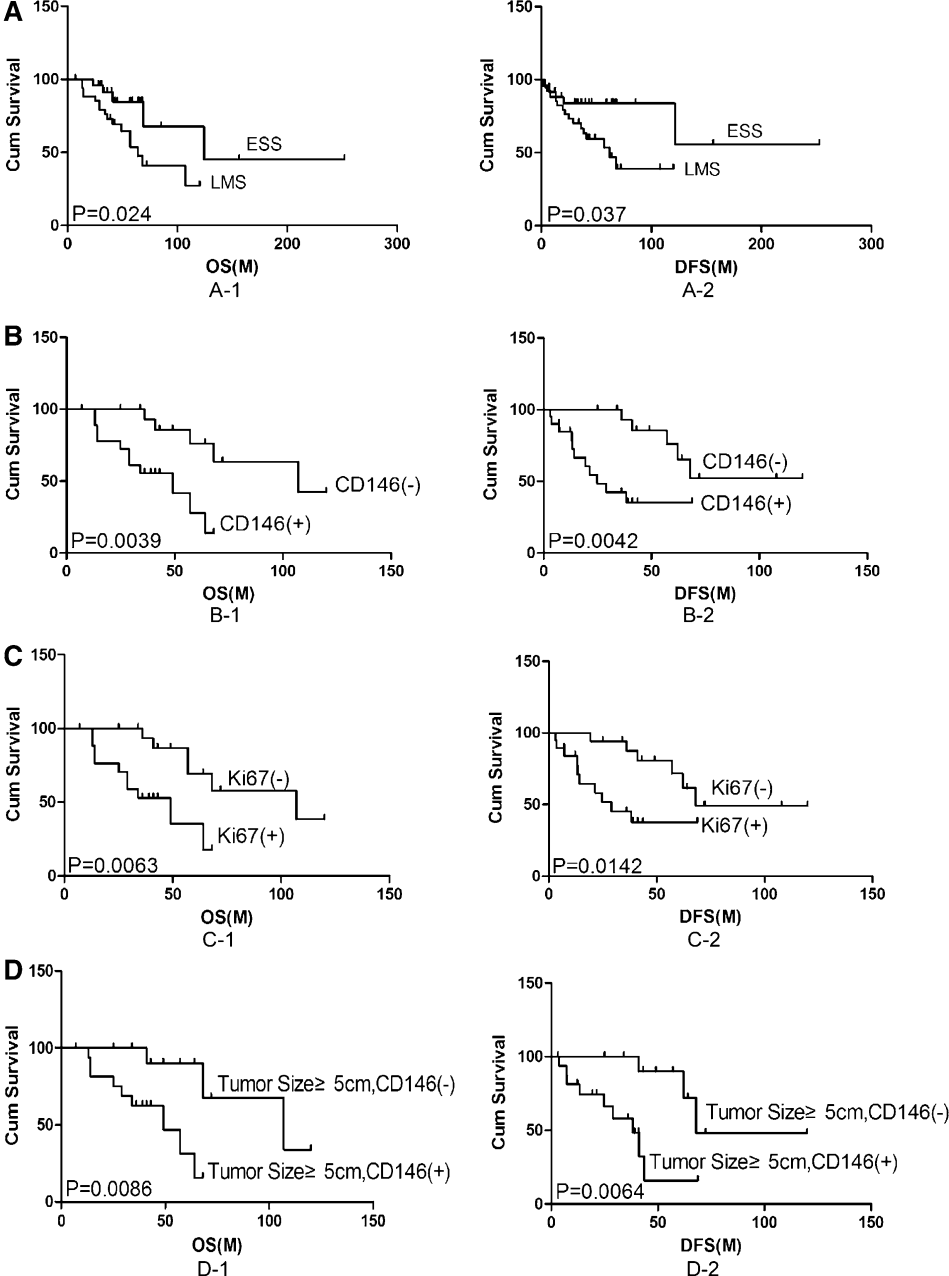
Kaplan–Meier curves obtained from survival analyses (log-rank) of patients with uterine sarcoma. **A** Histologic type was a significant prognostic factor for overall survival in LMS and ESS (*P* = 0.024). **B** CD146 showing significant correlation with overall survival in LMS. CD146-positive patients had a poor prognosis compared with CD146-negative patients (*P* = 0.0039). **C** Ki67 showing significant correlation with overall survival in LMS. Ki67-positive patients had a poor prognosis compared with Ki67-negative patients (*P* = 0.0063). **D** CD146 expression may be correlated with tumor size and poor prognosis in LMS (*P* = 0.026)

Even in more advanced-stage patients, the ESS group had better OS and DFS than the LMS group (Fig. 2A). The 2-year OS rate for ESS and LMS patients was 82 and 66 %, respectively. The median OS for ESS and LMS patients was 136.5 and 61.02 months, respectively (*P* = 0.024). The median DFS for ESS and LMS patients was 135.6 and 24.6 months, respectively (*P* = 0.037).

The median survival of patients with lymph node metastasis was 57 months compared with 136 months in lymph node-negative patients (*P* < 0.05). Tumor size ≥5 cm was a significant adverse prognostic factor for LMS (*P* = 0.02). The median survival of patients with tumor size <5 or ≥5 cm was 100 and 40 months, respectively. MI >10/HPF tended to be associated with an unfavorable outcome in LMS (*P* = 0.1). Multivariate analyses showed no statistical differences among these factors.

Adjuvant therapy had little impact on the management of uterine sarcoma, especially in early-stage disease. Postoperative chemotherapy did not improve the survival time of stage I ESS patients (*P* = 0.42).

### Prognostic significance of CD146, P53, and Ki-67 expression

CD146-positive LMS patients had a poorer prognosis than those who were CD146 negative. The 2-year OS rate was 53 % for CD146-positive patients and 84 % for CD146-negative patients (*P* = 0.0039, Fig. 2B). On multivariate analyses, CD146 positivity varied statistically significantly with clinicopathological parameters, P53, and Ki-67 expression (*P* = 0.01).

Ki-67 overexpression was also related to an unfavorable outcome in LMS, with a significant difference observed (*P* = 0.0063, Fig. 2C). P53 overexpression in LMS showed no significant difference (*P* = 0.1).

In LMS patients with tumor size ≥5 cm, Kaplan–Meier estimates yielded a significant survival difference favoring CD146-positive patients (*P* = 0.0086, Fig. 2D).

CD146 positivity also tended to be associated with poor prognosis in ESS, though the difference was not significant (*P* = 0.1). Similarly, P53 and Ki-67 overexpression in ESS were related to poor prognosis but the differences were not significant (*P* = 0.1).

## Discussion

Uterine sarcomas display aggressive clinical behavior and have a poor prognosis [3]. Our findings are in line with another study [17] in that the most common histologic type of uterine sarcoma detected was LMS (52.9 %) whereas MMMT accounted for only 10.3 % of cases. The median survival time of the 68 patients was 30.7 months; the 5-year OS and DFS rates were 46 and 44 %, respectively, which is consistent with a previous report in which OS and DFS were 45–47 and 34–36 %, respectively [17]. In a previous study, the outcome of ESS was significantly better than that of LMS and MMMT [18]. However, other studies have found, via multivariate analyses, that the histologic subtype does not affect survival. In our study, ESS patients had a better prognosis than those with other histologic subtypes. Even in more advanced-stage patients, the ESS group had better OS and DFS than the LMS group, with a 2-year survival rate of 82 %. A possible reason is that ESS tends to present as a less aggressive, early-stage disease with low levels of metastasis [18].

Previous studies have found that stage, surgical treatment, tumor grade, tumor size, and MI are prognostic factors for uterine sarcoma [19, 20]. Our results suggest that tumor size ≥5 cm, lymph node metastasis, and MI are significant prognostic factors in LMS. In our study, 38 of 68 cases (55.9 %) of primary uterine sarcoma expressed CD146 in the epithelial compartment. In LMS, CD146 expression in the epithelial compartment was associated with lymph node metastasis and was related to poor OS. Conversely, CD146 positivity in ESS patients did not correlate with OS. CD146-positive LMS had a poorer prognosis, the 2-year OS rate being 53 % compared with the 2-year OS of 84 % in CD146-negative LMS (*P* = 0.0039). In LMS patients with tumor size ≥5 cm, the outcome was worse for those who were CD146 positive (*P* = 0.0086). Similar to other carcinomas, CD146 was overexpressed and associated with tumor metastasis and poor prognosis [11, 21]. Aldovini et al. demonstrated that CD146 is involved in ovarian cancer and is a marker for poor prognosis in this disease [21].

CD146 is a 113 kDa integral membrane glycoprotein, the amino acid sequence of which comprises a signal peptide, an extracellular fragment structure of five immunoglobulin-like domains (V-V-C2-C2-C2), a transmembrane region, and a short cytoplasmic tail [22]. It has been suggested that CD146 promotes tumor growth, angiogenesis, and metastasis [10]. CD146 plays a critical pro-migratory role in the vascular system, normal development, and tumor progression patterning [23].

Interestingly, in the present study, CD146 was expressed in the vascular compartment in 49 cases, but also in the epithelial compartment in 38 cases. It has been established that LVSI is a significant prognostic factor in uterine sarcoma; LVSI and lymph node metastasis were shown to be relevant prognostic factors with an impact on OS and distant metastasis-free survival [24]. Moreover, on multivariate analysis, we found a significant relationship between CD146 overexpression and prognosis (*P* = 0.01). This implies that CD146 overexpression in the epithelial compartment may be related to angiogenesis and metastasis with a worse prognosis in LMS, and may serve as a potential LMS prognostic marker.

The prognosis of MMMT and UD may be related to CD146 expression in the vascular compartment. Due to the limited number of cases, we were unable to obtain significant statistical differences. The correlation between CD146 expression and clinicopathologic characteristics was not statistically significant.

CD146 positivity in the epithelial compartment showed a significant association with poor prognosis in ESS (*P* = 0.1). Likewise, P53 and Ki-67 overexpression in LMS were related to an unfavorable outcome, though no significant difference was observed (*P* = 0.1). One possible reason may be a lack of test power, as the sample size was small, or it could have been due to the different mechanism of progression and metastasis in ESS.

## Conclusions

CD146, P53, and Ki-67 were overexpressed in uterine sarcoma. CD146 expression in the epithelial compartment was correlated with lymph node metastasis and was associated with poor OS in LMS, but had no impact on survival in ESS. CD146 may be a potential prognostic marker for LMS.

## Abbreviations

WHO: World Health Organization
LMS: leiomyosarcoma
ESS: endometrial stromal sarcoma
UD: undifferentiated sarcoma
MMMT: malignant mixed Müllerian tumor
SYSUCC: Sun Yat-Sen University Cancer Center
FIGO: International Federation of Obstetrics and Gynecology
PE: paraffin-embedded
TAH: total abdominal hysterectomy
BSO: bilateral salpingo-oophorectomy
PL: pelvic lymphadenectomy
MI: mitotic index
HPF: high-power field
OS: overall survival
DFS: disease-free survival
LVSI: lymphovascular space invasion
